# A glimpse of the connection between PPARγ and macrophage

**DOI:** 10.3389/fphar.2023.1254317

**Published:** 2023-08-28

**Authors:** Lexiang Yu, Yuen Gao, Nicole Aaron, Li Qiang

**Affiliations:** ^1^ Naomi Berrie Diabetes Center, Columbia University, New York, NY, United States; ^2^ Department of Pathology and Cell Biology, Columbia University, New York, NY, United States; ^3^ Department of Medicine, Vagelos College of Physicians and Surgeons, Columbia University, New York, NY, United States; ^4^ Department of Physiology, Michigan State University, East Lansing, MI, United States; ^5^ Department of Molecular Pharmacology and Therapeutics, Columbia University, New York, NY, United States

**Keywords:** macrophage, antagonists, inflammatory diseases, PPARγ, anti-inflammatory

## Abstract

Nuclear receptors are ligand-regulated transcription factors that regulate vast cellular activities and serve as an important class of drug targets. Among them, peroxisome proliferator-activated receptors (PPARs) are members of the nuclear receptor family and have been extensively studied for their roles in metabolism, differentiation, development, and cancer, among others. Recently, there has been considerable interest in understanding and defining the function of PPARs and their agonists in regulating innate and adaptive immune responses and their pharmacological potential in combating chronic inflammatory diseases. In this review, we focus on emerging evidence for the potential role of PPARγ in macrophage biology, which is the prior innate immune executive in metabolic and tissue homeostasis. We also discuss the role of PPARγ as a regulator of macrophage function in inflammatory diseases. Lastly, we discuss the possible application of PPARγ antagonists in metabolic pathologies.

## 1 Introduction

Peroxisome proliferator-activated receptors (PPARs) are transcription factors that rely on ligands for their activation. They belong to the nuclear receptor superfamily and share a conserved structure. In mammals, there are three types of PPARs: PPARα, PPARδ (sometimes referred to as PPARβ), and PPARγ, also known as NR1C1, NR1C2, and NR1C3, respectively (Kliewer et al., 1994; Chawla et al., 2001b). Each type is encoded by a separate gene located on a different chromosome. PPARs are expressed in various tissues and cell types, influencing several cellular functions such as proliferation, differentiation, glucose and lipid metabolism, insulin signaling, inflammation, and tumorigenesis, among others (Chawla et al., 1994; Giusti et al., 2003; Odegaard et al., 2007; Harmon et al., 2011). PPARγ is the most extensively studied among the PPAR types. It comprises of four main domains: a ligand-dependent transcriptional activation domain (A/B domain) at the N-terminus, a DNA-binding domain (C domain), a hinge region (D domain), and a ligand-binding domain (E/F domain) at the C-terminus (Escher and Wahli, 2000). PPARγ is highly conserved in humans and mice, sharing 96% homology in amino acid sequences. The open reading frame (ORF) of the PPARγ gene in both species consists of six exons. Exons 2 and 3 encode the DNA-binding domain, while exons 5 and 6 encode the ligand-binding domain (Chen et al., 2006). PPARγ has two isoforms, γ1 and γ2, generated from alternate promoter usage and differential splicing with different cell expression pattern (Zhu et al., 1995). For example, PPARγ1 is the dominant form in macrophages and actually broadly expressed; in contrast, PPARγ2 is mostly restricted in adipocytes, regulating almost every aspect of adipocyte biology (Berger and Moller, 2002). Given their different expression pattern and function, PPARγ1 and PPARγ2 should be more carefully distinguished.

The significance of PPARγ in immune cells, particularly macrophages, has been well established (Chawla, 2010). Macrophages are specialized cells derived from bone marrow, playing crucial roles in tissue homeostasis and the innate immune response. Macrophage activation is essential for the innate immune response and serves as the initial defense against disruptions in tissue homeostasis. Dysregulation of macrophage activities is closely linked to the development of chronic diseases such as obesity, atherosclerosis, aging, fibrosis, and cancer. Macrophages within different organs possess specific functions dictated by tissue heterogeneity (Wculek et al., 2022).

PPARγ has been demonstrated to regulate the key activities in macrophages, including differentiation, inflammatory activation, polarization, and lipid metabolism. This review is focused on the recent progress of PPARγ function in macrophages and the connection with immunometabolism. In addition, we highlight some understudied directions in metabolism and cellular communication.

## 2 Transcriptional mechanisms of PPARγ

**FIGURE 1:**
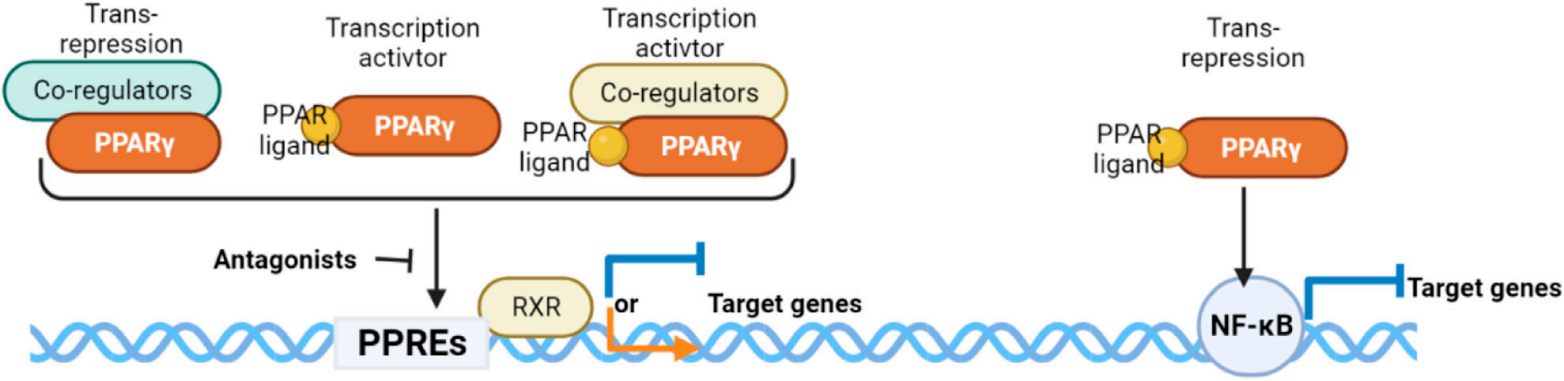
Mechanisms of action exerted by the PPARγ/RXRα heterodimer.

PPARγ exerts its regulatory influence on multiple metabolic and inflammatory signaling pathways by controlling the transcriptional activity of target genes. This regulation occurs through direct binding to PPAR response elements (PPREs) located on the promoters of specific genes, either in a ligand-dependent or ligand-independent manner (Figure 1). This binding event modifies the structure of chromatin and facilitates the recruitment of various cofactors, including coactivators and corepressors, which ultimately govern gene expression (Glass and Ogawa, 2006). PPARγ primarily forms a heterodimeric complex with the retinoid X receptor (RXR) to enhance transcriptional activity (Li et al., 2000; Zhang et al., 2002) positively regulates the expression of target genes or to exert transrepressive effects (Kamei et al., 1996) negatively regulates gene expression, effectively repressing the activity of certain genes. These versatile functions of PPARγ allow it to regulate an extensive network of genes involved in lipid metabolism and glucose homeostasis across diverse tissues such as adipose tissue, muscle, liver, and others. While the exact nature of endogenous ligands for PPARγ *in vivo* is still not well characterized, they are believed to be derivatives of fatty acids that are produced locally through paracrine or autocrine mechanisms (Kliewer et al., 1997; Huang et al., 2019b). Notably, various fatty acid metabolites generated during the inflammatory response can activate PPARs, and macrophages play a significant role in the production of endogenous ligands for PPARγ (Glass and Saijo, 2010).

## 3 Macrophage activation

Macrophages are crucial innate immune cells with a phagocytic function that involves clearing microorganisms, apoptotic and necrotic cells, and contributing to tissue remodeling (Gordon et al., 2014). The functional diversity of macrophages is primarily manifested through their distinct roles governed by signaling factors, metabolic changes (Glass and Natoli, 2016) and crosstalk (Sárvári et al., 2021; Ren et al., 2023). Historically, activation studies have predominantly focused on primary macrophages and macrophage cell lines, which were exposed to single polarizing ligands such as lipopolysaccharide (LPS), interferon gamma (IFNγ), or interleukin 4 (IL-4) *in vitro* (Glass and Natoli, 2016). These ligands trigger signaling cascades upon engaging cell surface receptors. For instance, in response to invading pathogens and Th1 cytokines like IFNγ, macrophages assume a proinflammatory immune response (known as the proinflammatory/classical activation state, M1), which involves pathogen phagocytosis (Gordon, 2003; Gosselin et al., 2014). Conversely, in the presence of Th2 cytokines like IL-4 and IL-13, macrophages adopt an anti-inflammatory immune tolerance state (termed the anti-inflammatory/alternative activation state, M2) to facilitate tissue repair processes (Gordon, 2003; Gosselin et al., 2014; Toobian et al., 2021). Apart from cell surface receptor activation, macrophage phenotypes are significantly influenced by intracellular and extracellular signals that are regulated by members of the nuclear receptor superfamily. Notably, PPARγ, glucocorticoid receptor, and liver X receptor (LXR) act as counter-regulators, inhibiting the transcriptional activity of proinflammatory transcription factors, including NF-κB, through direct and indirect mechanisms (Glass and Saijo, 2010; Li et al., 2013; Ito et al., 2015). These intricate regulatory networks determine macrophage distinct activity states and impose their tissue-specific properties.

Macrophages in different activation states exhibit corresponding gene expression profiles. The proinflammatory state is characterized by the presence of immunoreactive cytokine markers such as NOS2, TNFα, IL-6, IL-1β, and MCP1. In contrast, macrophages in an immune tolerance state express anti-inflammatory markers including CD36, IL-13, Arg1, Ym1, Fizz1, CD206, IL-4, and IL-10. The expression of these markers is tightly coordinated. For instance, treatment with LPS induces the typical proinflammatory activation of mouse macrophages, upregulating Th1 cytokines such as TNF-α, IL1-β, IL-6, and IL-12 while downregulating Th2 cytokine IL-10 (Shapouri-Moghaddam et al., 2018). Macrophages possess heterogeneity to maintain a balance between proinflammatory and anti-inflammatory immune states to function appropriately. Impaired switching between these two states is implicated in tissue damage and the development of chronic diseases.

## 4 The connection between macrophage and PPARγ

Activated macrophages are multifaceted immune cells that play crucial roles in both innate and adaptive immunity. They can present processed antigens to T cells, while activated T cells, in turn, secrete cytokines that further activate macrophages (Guerriero, 2019). Growing evidence suggests that PPARγ also plays a significant role in the immune system. In addition to its role as a master regulator of adipocyte differentiation, PPARγ is induced during the differentiation of monocytes into macrophages. It is expressed on various immune cells, including monocytes/macrophages, dendritic cells (DCs), T and B lymphocytes, and platelets (Padilla et al., 2000; Gosset et al., 2001; Setoguchi et al., 2001; Alleva et al., 2002; Rotondo and Davidson, 2002). However, PPARγ-deficient embryonic stem cells have been shown to differentiate into macrophages (Chawla et al., 2001b). PPARγ also influences the differentiation of fetal monocytes into alveolar macrophages (Ginhoux, 2014).

**FIGURE 2:**
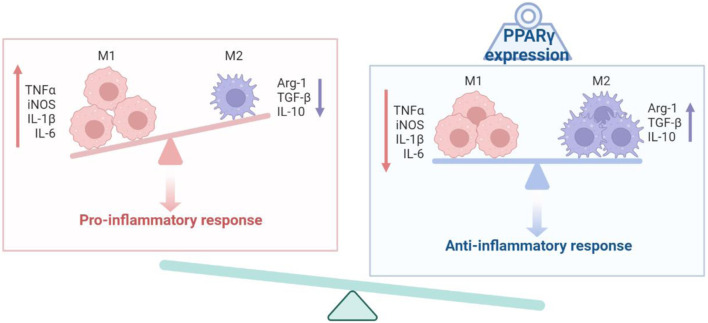
Schematic diagram of the PPARγ and the activation state of macrophages.

PPARγ activation has been reported to suppress the immune response of macrophages (Figure 2). In the absence of PPARγ, mouse macrophages exhibited upregulation of proinflammatory levels and downregulation of anti-inflammatory cytokines when induced with LPS (Heming et al., 2018). Additionally, PPARγ inhibits the expression of HIF1a, a crucial regulator of immune responsiveness, thereby increasing the expression of arginase 1, an anti-inflammatory marker (Blum et al., 2016). PPARγ exerts its effects on immune cells by directly activating the transcription of target genes through DNA binding. Moreover, IL-4/STAT6 signaling and IL-13 induce PPARγ expression, with STAT6 or PSTPIP2 acting as a “facilitator” of PPARγ signaling, resulting in the promotion of anti-inflammatory responses (Huang et al., 1999; Xu and Lv, 2023). Notably, specific anti-inflammatory genes, such as Arg1 and Mgl1, are identified as direct PPARγ target genes (Gallardo-Soler et al., 2008). These findings indicate that PPARγ not only influences the immune state of macrophages but also plays a role in regulating the overall metabolic states (Bouhlel et al., 2007; Odegaard et al., 2007). *In vivo* studies have also shown the response of PPARγ to infection. Macrophages are involved in post-infection repair and the clearance of immune antigenic fragments. Activation of PPARγ increased Fcg receptor-mediated phagocytosis in murine alveolar macrophages, indicating its role in regulating phagocytic clearance (Aronoff et al., 2007). PPARγ-deficient mice have consistently shown impaired skin wound healing due to defective clearance of apoptotic cells (Chen et al., 2015). PPARγ has also been implicated in proper tissue repair after influenza infection, as PPARγ-deficient mice exhibited increased collagen deposition in the lungs (Huang et al., 2019a). Furthermore, PPARγ has demonstrated its ability to promote the macrophage phenotype of immune tolerance.

Apart from its direct effects on insulin sensitization and adipocyte development, PPARγ plays a role in macrophages present in adipose tissue, skeletal muscle, and liver. PPARγ, functioning as a metabolic sensor and transcriptional regulator, governs the expression of GDF3, a member of the transforming growth factor-β (TGF-β) family. GDF3, in turn, plays a crucial role in restoring skeletal muscle integrity by promoting the fusion of muscle progenitor cells, thereby mediating the regenerative effects of specialized macrophage-derived factors in tissue repair (Hevener et al., 2007; Varga et al., 2016). Macrophages also infiltrate adipose tissue, facilitating the mobilization of macrophages from the bone marrow into adipose tissue (mediated through MCP1) and efficiently clearing impaired adipocytes (Kanda et al., 2006). Additionally, macrophage migration is induced by localized microhypoxic regions in adipose tissue (Hif1a) (Lolmède et al., 2003; Trayhurn and Wood, 2004; Hosogai et al., 2007), contributing to hepatic fatty acid shunting processes during fasting conditions (Nguyen et al., 2005; Suganami et al., 2005; Shi et al., 2006). Post-infiltration, macrophages display significant heterogeneity in their activity and function, reflecting changes in metabolic and immune disturbances (Gordon and Taylor, 2005). Central in the pathogenesis of chronic liver injury, hepatic macrophages are a highly heterogeneous population of immune cells that perform multiple functions in homeostasis, disease progression, and resolution of injury (Zimmermann et al., 2012; Tacke and Zimmermann, 2014). Furthermore, macrophages help to clear pathogens or cellular debris and to maintain immune tolerance under homeostatic conditions (Fallowfield et al., 2007), promoting hepatic fibrosis through activation of hepatic stellate cells in chronic liver damage (Imamura et al., 2005; Mitchell et al., 2009), ultimately underscoring their central role in tissue response to injury and inflammation (Miura et al., 2012).

In addition to macrophage infiltration into tissue, bone formation (Stechschulte et al., 2016), bone resorption (Stechschulte et al., 2016), anti-atherogenesis (Grbić et al., 2018; Liu et al., 2020a; Zahr et al., 2022), inflammation (Yang et al., 2016), metabolic rhythmicity (He et al., 2022), lung fibrotic sequelae (Huang et al., 2019a), and adipose tissue browning (Qiang et al., 2012) are regulated by PPARγ post-translational modifications. Multiple factors regulate PPARγ expression levels, which may affect the polarization of macrophages, and thus affect the disease process (Doyle et al., 2002; Gu et al., 2020; Li et al., 2020; Chen et al., 2021).

## 5 PPARγ related signaling pathways in macrophage regulation

Activated macrophages express significant levels of PPARγ (Chawla et al., 2001b). The absence of PPARγ signaling leads to continued secretion of high levels of proinflammatory cytokines by macrophages (Bouhlel et al., 2007), indicating that PPARγ may have multiple effects on macrophage state. Studies have demonstrated that PPARγ agonists act as negative regulators of monocytes and macrophages, inhibiting the production of proinflammatory cytokines such as TNFa, IL-1b, and IL-6 (Jiang et al., 1998; Reddy, 2008). Moreover, the PPARγ agonist rosiglitazone has been shown to suppress the expression of proinflammatory cytokines induced by LPS, particularly at higher concentrations (Welch et al., 2003).

Inflammation and oxidative stress caused by macrophages are common pathological processes that accompany, promote and even trigger various cancers or various chronic metabolic diseases, such as aging, obesity, Alzheimer’s disease, etc. Many of them involved in Wnt/β-catenin, p-JNK, p-AKT, AMPK/eNOS, etc (Khansari et al., 2009). 1) Activation of the canonical Wnt/β-catenin pathway induces PPARγ inactivation, whereas PPARγ activation induces inhibition of canonical Wnt/β-catenin signaling. The canonical Wnt/β-catenin pathway is increased while PPARγ is downregulated in pathogenesis (Vallée et al., 2018). 2) PPARγ-dependent mechanism downregulates cardiovascular, inflammatory markers through p-JNK signaling (Mohanty et al., 2004). 3) TZDs prevent cardiomyocyte apoptosis through PPARγ ligand-dependent induction of upregulation of AKT phosphorylation (Kilter et al., 2009). 4) Activation of PPARγ induces phenotypic changes from M1 to M2 in macrophages at sites of inflammation through a heme oxygenase 1 (HO-1)-dependent mechanism (von Knethen et al., 2011). 5) Evidence that activation of PPARγ by TZDs regulates muscle and cardiac glucose metabolism through AMPK (Ye et al., 2006) and AMPK/eNOS phosphorylation (Xiao et al., 2010), respectively.

## 6 PPARγ and cholesterol metabolism in macrophages

Monocytes migrate to the arterial wall and undergo differentiation into macrophage “foam cells,” characterized by the accumulation of cholesteryl esters, a key feature of early and advanced atherosclerotic lesions. The uptake of modified forms of Low-density lipoprotein (LDL), particularly through scavenger receptors (SR-A), is believed to mediate cholesterol accumulation in macrophages (Krieger and Herz, 1994; de Villiers and Smart, 1999). SR-A recognizes acetylated and oxidized LDL, while CD36 exhibits greater selectivity for oxidized LDL (oxLDL), resulting in reduced uptake and degradation by macrophages from CD36-null mice compared to control macrophages (Podrez et al., 2000). Given the strong expression of PPARγ in macrophages within atherosclerotic plaques (Ricote et al., 1998), it was hypothesized that pharmacological activation of PPARγ could reduce plaque inflammation and impairments. Supporting this hypothesis, the Evans lab demonstrated that components of oxLDL, specifically 9- and 13-hydroxyoctanoic acid (HODE), transcriptionally activate PPARγ, implicating PPARγ in promoting atherogenesis (Nagy et al., 1998; Tontonoz et al., 1998). Consequently, activation of the LXR/RXR heterodimer via the PPARγ pathway upregulates the expression of ApoE, ABCA1, and SREBP1c, facilitating cholesterol outflow in macrophages and reinforcing reverse cholesterol transport to diminish lipid accumulation in macrophages (Janowski et al., 1996; Li et al., 2004).

The effects of PPARγ activation vary depending on the nature of the activator. Notably, exposure of macrophages to oxLDL has been reported to activate PPARγ in a PKC-dependent manner (Nagy et al., 1998; Feng et al., 2000). To date, CD36 has been identified as the canonical macrophage gene directly regulated by PPARγ, leading to enhanced CD36 expression and subsequent stimulation of oxLDL uptake (Febbraio et al., 2000; Rahaman et al., 2006). Consequently, exogenous activation of the PPARγ ligand-dependent pathway may promote CD36 expression and oxLDL uptake. However, in PPARγ-null macrophages, the loss of CD36 regulation does not significantly alter lipid uptake, suggesting that PPARγ-CD36 does not solely govern the pathway for oxLDL uptake (Chawla et al., 2001a). Furthermore, PPARγ can enhance cholesterol efflux from cells by inducing the expression of LXRα.

## 7 Therapeutic potential and challenges associated with modulating PPARγ

Modulating PPARγ in macrophages holds significant therapeutic promise for various diseases. Which are involved in anti-inflammatory, metabolic disorders, immunomodulation and tissue repair and regeneration. Activating PPARγ can shift macrophage polarization towards the anti-inflammatory M2 phenotype, dampening excessive inflammation and promoting tissue repair (Eming et al., 2017; Nelson et al., 2018; Abdalla et al., 2020; Wculek et al., 2022). This approach shows potential in treating chronic inflammatory conditions like rheumatoid arthritis, inflammatory bowel disease, and metabolic disorders such as diabetes and dyslipidemia (Gu et al., 2014; Jalil et al., 2014; Baillie et al., 2017; Na et al., 2019; Cataldi et al., 2021). Additionally, PPARγ activation can modulate macrophage phagocytosis, antigen presentation, and cytokine production, influencing the overall immune response (Yu et al., 2016; Ciavarella et al., 2020; Mierzejewski et al., 2022). Harnessing these immunomodulatory effects may hold potential in immunotherapeutic strategies and cancer treatment, as macrophages play a critical role in tumor microenvironments.

However, challenges need to be addressed for successful clinical applications. One such challenge is the off-target effects: PPARγ is widely expressed in various tissues, and its modulation may have unintended effects on other cell types beyond macrophages (Ahmadian et al., 2013). These off-target effects could lead to adverse reactions and complicate treatment strategies. Furthermore, the context-specific nature of PPARγ modulation presents complexities, as therapeutic outcomes may vary depending on the disease context and microenvironment (Kung and Henry, 2012). Tailored approaches for different diseases may be necessary to optimize treatment efficacy (Mayerson et al., 2002). Patient heterogeneity also poses a challenge, as individual responses to PPARγ modulation may be influenced by genetic and environmental factors (Liu et al., 2020b). Implementing personalized medicine approaches will be essential to maximize treatment benefits and minimize potential risks.

In conclusion, targeting PPARγ in macrophages presents exciting therapeutic possibilities for various diseases. However, addressing the challenges, such as off-target effects, drug-specific effects, and patient heterogeneity, is essential to realize the full potential of PPARγ modulation for effective and safe clinical applications. Continued researches and clinical trials are necessary to unravel the complexities and refine the use of PPARγ modulation in the context of macrophages for precision medicine approaches.

## 8 PPARγ antagonists and their therapeutic potential

Considering the high expression of PPARγ in the pro-inflammatory state and the partial anti-inflammatory properties of PPARγ activation, PPARγ seems to play a role in the regulation of macrophage lipid metabolism in activated macrophages. Overactivation and persistent chronic inflammation are major pathogenic features of impaired healing in multiple metabolic diseases, such as diabetes, multiple sclerosis, SARS-CoV-2 et, al. (Yu et al., 2019; Jabbari et al., 2021; Kumar et al., 2021; Eleftheriotis et al., 2023).The overactivation of phagocytes can be inhibited by PPARγ antagonists, in addition to rebalancing the lipid metabolism and glucose metabolism of macrophages, it also improves the pro-inflammatory state of macrophages in the immune tolerant state (Toobian et al., 2021), and PPARγ was also regard as modulator of inflammation in pulmonary sarcoidosis (Pejcić et al., 2013). The use of PPARγ antagonists is also a novel therapeutic strategy being explored, for example, as it pertains to the ability of PPARγ antagonists to regulate lipid metabolism in mouse models of type 2 diabetes (T2DM), like the Gleevec, a renowned anticancer drug, acts as a PPARγ ligand without classical agonism, inhibiting PPARγ phosphorylation at S273 (Rieusset et al., 2002; Burton et al., 2008; Wang et al., 2015; Choi et al., 2016), cervical cancer (An et al., 2014), and to inhibit adipose tissue differentiation (Wright et al., 2000). Additionally, PPARγ antagonists are considered a potentially novel oncology therapeutic because of their antiproliferative effects on cancer cells (Burton et al., 2008). Furthermore, there is a vital link between fatty acid metabolism and tumorigenesis (Hoy et al., 2021), especially in adipose tissue organ-related breast cancer (Seargent et al., 2004; Zaytseva et al., 2011). Of most interest, PPARγ antagonists (GW9662) play a role in macrophage differentiation, regulating the expression of apoptosis-phagocytosis genes in apoptotic cells (Seargent et al., 2004; Majai et al., 2007) and inhibits growth of breast tumour cells (Seargent et al., 2004), which support a PPARγ-mediated transrepression mechanism, previously shown to be responsible for the anti-inflammatory effects of PPARγ ligands through the NF-κB signaling pathway (Pascual et al., 2005). As previously mentioned, immunosuppressive macrophages functions include post-infection repair and clearance of debris (Cui and Ferrucci, 2020). Additional studies below elaborate on PPARγ antagonists and their related application (Table 1).

**TABLE 1:** PPAR antagonists in development.

PPARγ antagonist	Indication	Functional related	Status	Reference
GW9662	Cancer, T2DM, Obesity	Tumor growth inhibition, Cell differentiation and apoptosis-phagocytosis	Preclinical	Rieusset et al. (2002), Seargent et al. (2004), Majai et al. (2007)
T0070907	Cervical cancer	Increase G2/M phase cell ratio	Preclinical	Zaytseva et al. (2011), An et al. (2014)
Mifobate (SR-202)	Obesity, T2DM	Improve adipocyte hypertrophy and insulin resistance	Phase II clinical trials	Rieusset et al. (2002)
Bisphenol A diglycidyl ether (BADGE)		Adipocyte differentiation, PPARγ ligand		Wright et al. (2000)
N-((1H-benzo[d]imidazol-2-yl)methyl) aniline (Compound Q)		Reduce RXRa-PPARγ heterodimer activity		Wang et al. (2015)
Betulinic acid	HIV, malaria dysplastic, nevus syndrome, melanoma	Induces apoptosis, Increases ROS and caspase activation	Phase II clinical trials	Huang et al. (2018), Jiang et al. (2021), Oliveira-Costa et al. (2022)
Gleevec	Leukemia, T2DM	Inhibits tyrosine kinase, Improve insulin resistance	Approved	Lin et al. (2013), Choi et al. (2016)

## Conclusion

Over the past decade, much has been learned about the function of PPARs in macrophages. While initial studies focused on the transcriptional mechanisms by which PPARγ may regulate cholesterol metabolism in macrophages, recent work has elucidated several novel regulatory roles for PPARγ during pathological changes. Furthermore, the elucidation of macrophage activation and diverse signaling pathways provides multiple possible explanations for the mechanisms that integrate lipid signaling into macrophage activation. The in-depth studies performed to date on the PPARγ-macrophage mechanism will provide guidance for the application and development of PPARγ antagonists as well as potential regulation of other nuclear receptors.
